# Diagnostic value of *N*-acetyl-β-D-glucosaminidase for the early prediction of acute kidney injury after percutaneous nephrolithotripsy

**DOI:** 10.3892/etm.2012.737

**Published:** 2012-10-08

**Authors:** CHEN JIANG, CHEN QI, KAI SUN, LEI XIA, WEI XUE, YIRAN HUANG

**Affiliations:** Department of Urology, Renji Hospital, Shanghai Jiao Tong University School of Medicine, Shanghai 200127, P.R. China

**Keywords:** acute kidney injury, *N*-acetyl-β-D-glucosaminidase, percutaneous nephrolithotripsy

## Abstract

The present observational study was undertaken in order to evaluate the diagnostic value of urinary *N*-acetyl-β-D-glucosaminidase (NAG) for the prediction of acute kidney injury (AKI) in patients after percutaneous nephrolithotripsy (PNL). Pre- and post-operative patient data were collected for 90 patients who underwent PNL between September 2008 and December 2010. The patients included 64 males and 26 females with an average age of 52.8±9.7 years. Pre- and post-operative urinary NAG was measured by colorimetric assay and serum creatinine levels were determined for comparative analysis. Urinary NAG levels significantly increased after PNL compared to pre-operative levels (P<0.05). AKI occurred in 11 cases after surgery. A comparison of the AKI and non-AKI groups revealed no significant differences in age, gender ratio or baseline creatinine levels (P>0.05); however, there were significant differences between the groups as regards surgical duration, post-operative infection rate, C-reactive protein levels and number of hospital days (P<0.05). NAG levels were significantly higher in the AKI compared to the non-AKI group after surgery (P<0.05). The diagnostic utility of the increase in urinary NAG 24 h after surgery was assessed by receiver operating characteristic (ROC) analysis. For an increase in NAG of 235.44%, the area under the ROC curve was 0.878 (P<0.01) and the sensitivity and specificity for AKI diagnosis were 81.8 and 91.1%, respectively. Urinary NAG significantly increased in patients suffering from AKI after surgery. This parameter is more sensitive than serum creatinine and can reflect the impairment of kidney function at an earlier stage. The surgical duration and post-operative infection rate are possible risk factors for AKI. Urinary NAG may have some clinical value in the early diagnosis of AKI after surgery.

## Introduction

Percutaneous nephrolithotripsy (PNL) is replacing open surgery as the preferred treatment for kidney and upper ureter stones due to its minimal invasiveness, high probability of success, and low probability of mortality. However, retrospective analysis has revealed that certain patients undergoing unilateral PNL suffer from acute kidney injury (AKI) after surgery. AKI can lead to a series of clinical problems; if not found and treated in a timely manner, it can extend the length of hospital stay, affect recovery of kidney function, increase mortality and morbidity (1–3) and increase therapy costs (including hemodialysis as renal replacement therapy). Laboratory results have indicated that early treatment can effectively prevent pathophysiological progression to AKI (4,5).

In recent years, studies have provided strong evidence that urinary biomarkers are useful for early-stage AKI diagnosis and therapy, including *N*-acetyl-β-D-glucosaminidase (NAG) activity, neutrophil gelatinase-associated lipocalin, interleukin-18 and kidney injury molecule-1 (6). To date, no study has assessed the clinical utility of NAG in PNL patients. This study examined whether urinary NAG levels from the kidney on the surgical side can predict AKI after unilateral PNL.

## Patients and methods

### Patients

This was a single-center, retrospective study. Data were collected for patients undergoing PNL in our hospital between September 2008 and December 2010.

The inclusion criteria were as follows: i) clear diagnosis of a kidney stone; ii) normal results for kidney function, routine blood parameters, electrolytes, routine urine analysis and midstream urine culture; and iii) suitability for PNL, with no hypertension, diabetes or coronary heart disease and no recent intake of drugs that affect renal function. Patients with serious hydronephrosis, a serious disease, renal insufficiency, obesity, emaciation, renal ectopia, a horseshoe kidney or a functional or organic solitary kidney, or for whom incomplete clinical data or incomplete samples were available, were excluded from this study. The research was carried out according to the principles of the Declaration of Helsinki. Informed consent was obtained and the Shanghai Renji Hospital Ethics Committee approved the study. The patient data, which are contained within this article, were obtained by a hospital-based doctor at Shanghai Renji Hospital, Shanghai Jiao Tong University School of Medicine. Permission to use these data in this report has been obtained from all the subjects who participated in this study (7).

### Surgical procedure

All patients underwent unilateral PNL using an F18 channel. After successful air-intravenous anesthesia with the patient in a prone lithotomy position, a ureteroscope (Storz or Wolf) was inserted into the bladder and advanced into the ureter on the surgical side, led by a zebra wire. An F5 ureteral catheter was then inserted.

A puncture was made in the pelvis guided by B ultrasound and a 10 ml pre-operative urine sample was collected and stored at 4°C. The channel was expanded to F18 with a fascial dilator led by a guide wire. After the percutaneous-nephro passage was established, the kidney stone was located using the ureteroscope and was broken up using a holmium laser. The majority of broken stones can be washed out by the filling pump through the working sheath. Consistent filling pump flow and pressure were maintained and recorded.

The stones were removed from the pelvis. When the procedure was complete, the pressure measurement equipment was removed, and a retrograde guide wire and double-J pipe were inserted. A F16 nephrostomy tube was sutured in place to drain urine from the surgical side after surgery.

At 2, 4, 6, 12, 24, 48 and 72 h after surgery, 10 ml urine samples were collected from the kidney on the surgical side.

### Sample analysis

All urine samples were stored at 4°C and centrifuged for 3 min at 2,000 rpm within 1 week. The supernatant was used for NAG measurement.

Urinary NAG activity was measured using the kinetic rate assay (8) with 2-chloro-4-nitrophenol(-yl) (CNP)-NAG (Quark Biotechnology Research Institute, Cleveland, OH, USA) as the substrate. Reactions were carried out at 37°C and pH 4.8. The rate of CNP production was determined from the change in absorbance at 400 nm per minute on an Olympus AU640 automatic analyzer. NAG activity was then calculated as U/l. To eliminate the influence of urine amount on enzyme activity, results are reported as the ratio of enzyme activity to urinary creatinine (U/mmol).

Serum creatinine (Scr) levels were measured in a single laboratory on a Hitachi 7600 analyzer using Jaffe’s kinetic method, reference range 40–140 μmol/l.

Serum C-reactive protein (CRP) levels were measured by latex agglutination immunoassay using the Nanopia CRP kit (Daiichi Pure Chemicals, Tokyo, Japan). Normal values provided by the manufacturer were ≤0.30 mg/dl, and therefore patients with a serum CRP of ≤0.30 mg/dl were considered the ‘normal CRP’ cohort.

### Statistical analysis

The SPSS 11.5 package was used for all statistical analyses. For parametric variables with a normal distribution, the results are presented as the means ± SD and comparisons between two groups were carried out using a t-test. For parametric variables with a non-normal distribution, the results are presented as the median (interquartile range), and comparisons between groups were carried out using a rank test. The sensitivity and specificity of AKI diagnosis according to NAG levels were assessed by receiver operating characteristic (ROC) analysis. A value of P<0.05 was considered to indicate a statistically significant difference.

## Results

Data were collected for 115 patients. Of these, 25 were excluded due to incomplete samples or medical history. Thus, 90 patients were included in this study. They ranged in age from 35 to 72 years (52.8±9.7) and included 64 males and 26 females.

For all patients, urinary NAG increased 2 h after surgery to reach a peak at 6 h at twice the level prior to surgery, and then gradually decreased. Post-operative NAG levels were significantly higher at various time-points compared to pre-operative levels (P<0.05; Table I).

Patients were categorized as AKI or non-AKI according to the AKI diagnosis criterion of a sudden decrease in kidney function over 48–72 h, evident as an absolute increase in Scr levels ≥0.3 mg/dl (≥26.4 mmol/l) or a percentage increase of ≥50%. Parameters for the groups are compared in Table II.

**Figure 1:**
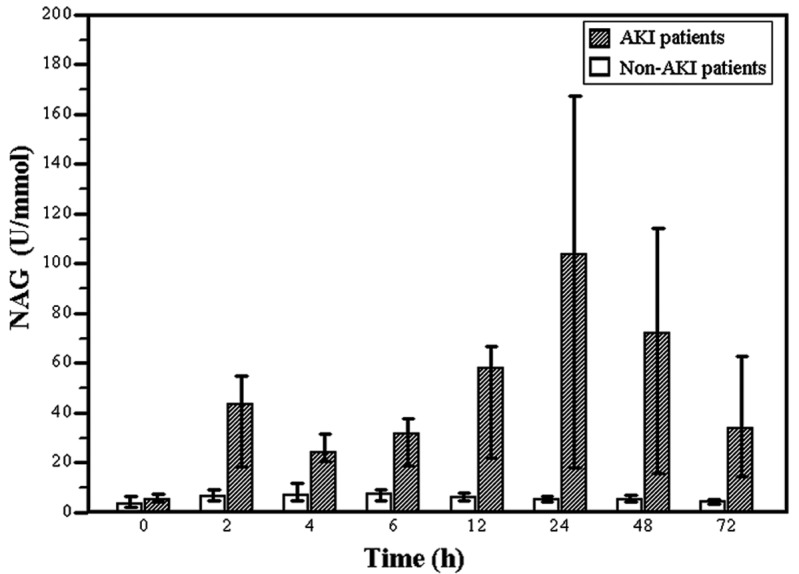
Comparison of *N*-acetyl-β-D-glucosaminidase (NAG) activities between acute kidney injury (AKI) and non-AKI patients. Levels in the AKI group increased at 2 h after surgery and were significantly higher compared to the non-AKI group (P<0.01).

There were no significant differences between the groups as regards age, gender ratio or baseline creatinine levels; however, there were significant differences in surgical duration, post-operative infection rate, CRP levels and number of hospital days (P<0.01; Table II). For the AKI group, NAG levels increased at 2 h after surgery and thereafter and were significantly higher compared to the non-AKI group (P<0.01; Fig. 1).

**Figure 2:**
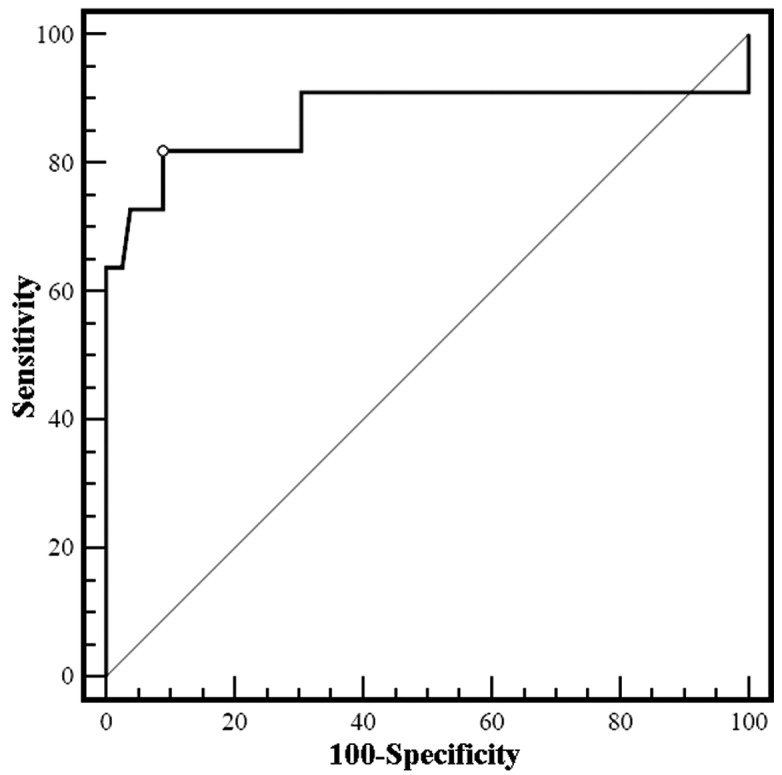
ROC curve of percentage of *N*-acetyl-β-D-glucosaminidase (NAG) increase for acute kidney injury (AKI) prediction. The area under the curve was 0.878 (95% CI, 0.699–1.043, P<0.01) for a NAG increase of 235.44%, with diagnostic sensitivity and specificity of 81.8 and 91.1%, respectively.

To assess the utility of the extent of the increase in urinary NAG at 24 h after surgery for AKI prediction, ROC analysis was carried out. The area under the curve was 0.878 (95% CI, 0.699–1.043, P<0.01) for a NAG increase of 235.44%, with diagnostic sensitivity and specificity of 81.8 and 91.1%, respectively (Fig. 2).

## Discussion

PNL as a surgical technique is minimally invasive; however, retrospective evaluation of a large amount of clinical data revealed that AKI occurs in some patients after unilateral PNL. Without timely adequate treatment, AKI can lead to an unfavorable prognosis. Early diagnosis and therapy are of great importance to reduce the risk of serious AKI complications.

The main index currently used in clinical practice for AKI diagnosis is Scr; however, its sensitivity and specificity are relatively low. Scr levels are only altered when renal function decreases by approximately 50% and Scr cannot reflect changes in renal function over time until the body reaches a stable condition, which often takes several days. Scr levels are influenced by other factors, such as age, race, blood volume, muscle metabolism, drugs and nutritional condition. These limitations mean that Scr is not an ideal index for AKI and cannot provide early clues for AKI diagnosis, so early clinical therapy may be delayed (9–14). Therefore, it is of clinical importance to identify biomarkers with higher sensitivity and specificity to predict, prevent and treat AKI after surgery.

In recent years, research on urine biomarkers has provided strong evidence that these are suitable for the early diagnosis and therapy of AKI (6). NAG has attracted increasing attention as such a biomarker. Widely distributed in various tissues and cells in the body, NAG is a lysosome hydrolase with a molecular weight of approximately 140,000 Da that cannot normally be filtered through the glomerulus (15). It is mainly distributed in lysosomes in epithelial cells of nephric tubules, and small amounts are found in the mitochondria. For patients with non-glomerulus disease without marked albuminuria, urinary NAG mainly originates from the nephric tubules. Since dynamic concentrations of urinary NAG change with urine flow and the collection of 24-h urine samples is complicated, the NAG/creatinine ratio has been used to avoid errors caused by random measurement at a single time-point. NAG is not easily inactivated in urine and urinary NAG output is relatively stable under normal conditions. Moreover, urinary NAG significantly increases during necrosis of the tubular epithelium and this change occurs much earlier than changes in blood urea nitrogen and Scr (16). Consequently, urinary NAG can be used to evaluate early damage to epithelial cells in the proximal convoluted tubules during the progression of renal diseases and is an index that reflects renal tubule damage (17).

Our results show that post-operative urinary NAG levels increased to different extents in 90 PNL patients, among whom 11 developed AKI. There were no significant differences between the non-AKI and AKI groups as regards age, gender ratio and baseline creatinine levels; however, the AKI group had a significantly longer surgical duration and a greater post-operative infection rate, CRP levels and number of hospital days. Thus, a longer surgical duration may increase the infection rate and AKI risk and prolong the hospital duration. If the surgical duration can be controlled to a certain extent, the risk of post-operative complications may be reduced.

In addition, NAG levels were higher in the AKI than in the non-AKI group within 24 h after surgery. Thus, NAG indicates the occurrence of AKI earlier and is a more sensitive biomarker than Scr. ROC analysis was carried out to assess the diagnostic utility of the increase in urinary NAG 24 h after surgery in predicting AKI. The area under the curve was 0.878 (95% CI, 0.699–1.043, P<0.01) for a post-operative increase in NAG of 235.44%, and the sensitivity and specificity of AKI diagnosis were 81.8 and 91.1%, respectively. Thus, the extent of any increase in urinary NAG levels may be an efficient marker for predicting AKI.

Currently AKI is divided into 3 phases (18). In phase I or the risk phase, Scr is >26.4 μmol/l (0.3 mg/dl) or increases by 50% and urine output has been <0.5 ml/(kg/h) for 6 h. In phase II or the damage phase, Scr increases by 200–300% and urine output has been <0.5 ml/(kg/h) for 12 h. In phase III or the exhaustion phase, Scr increases by more than 300% or is >4 mg/dl and urine output has been <0.3 ml/(kg/h) for 24 h or the patient has experienced anuresis for 12 h. During phase I, the treatment focus is on analysis and neutralization of risk factors, pathogen elimination, investigation of changes in daily intake and output volumes and body weight, evaluation of blood volume, and maintenance of electrolytes and acid-base balance. During phase II, the focus is on preventing or decreasing damage to target organs, to provide out-specific nursing care (nephrostomy tube, skin, psychology and fluid management), to identify any infection as early as possible, and to provide nutritional support. During phase III, as it is possible for renal function to recover completely or partially, the illness state is complicated, and clinical manifestations are various and unstable, dialysis should not be delayed until chronic renal failure occurs. On the contrary, preventive dialysis should be performed as early as possible to replace renal function, maintain body homeostasis and provide an opportunity for recovery of multi-organ function (19). Our AKI patients were classified as phase I, so the focus was as described above to prevent further deterioration of renal function. For AKI patients without other serious complications, treatment with drugs such as the selective dopamine receptor 1 agonist, fenoldopam, and the free radical eliminator and antioxidant, pentoxifylline, is feasible (20). Liangos *et al* found that urinary NAG positively correlated with the degree of acute renal failure (21). AKI detection at an early stage is important for enhanced vigilance and to prevent further worsening of AKI to phase III.

In conclusion, our results demonstrate that urinary NAG activity significantly increases in AKI. This parameter is more sensitive than Scr and can reflect impairment of renal function at an earlier stage. The surgical duration and post-operation infection rate are possible risk factors for AKI. Urinary NAG may have some clinical value in the early diagnosis of AKI after surgery.

## Figures and Tables

**Table I:** Urine NAG activities in the kidney on the surgical side at various time-points (U/mmol).

	0 h	2 h	4 h	6 h	12 h	24 h	48 h	72 h
Distance between median and interquartile range	3.82–4.20	7.19–7.58	7.73–7.71	8.11–6.12	6.565–4.45	5.79–2.82	6.20–2.90	4.78–3.39
P-value (compared to 0 h)		0.000	0.000	0.000	0.000	0.000	0.000	0.013

NAG, *N*-acetyl-β-D-glucosaminidase.

**Table II:** Comparison between AKI group and non-AKI group.

	AKI	Non-AKI	P-value
No. of cases	11	79	
Age	51.3±10.3	53.0±9.6	>0.05
Gender	8/3	56/23	>0.05
Surgical duration (min)	113.18±14.60	78.27±13.80	<0.01
Urinary passage infection rate (%)	81.82	37.97	<0.01
C-reactive protein (mg/l)	87.82±14.11	47.11±15.61	<0.01
Blood creatinine (μmmol/l)	51.96±15.32	58.73±11.14	>0.05
No. of hospital days	5.91±0.83	3.80±0.88	<0.01

AKI, acute kidney injury.
